# A Comprehensive Analysis of Human Endogenous Retroviruses HERV-K (HML.2) from Teratocarcinoma Cell Lines and Detection of Viral Cargo in Microvesicles

**DOI:** 10.3390/ijms222212398

**Published:** 2021-11-17

**Authors:** Vladimir A. Morozov, Alexey V. Morozov

**Affiliations:** 1 Department of Infectious Diseases, Robert Koch Institute, Nordufer 20, 13353 Berlin, Germany; 2 Laboratory of Regulation of Intracellular Proteolysis, Engelhardt Institute of Molecular Biology, Russian Academy of Sciences, 119991 Moscow, Russia; 3 Center for Precision Genome Editing and Genetic Technologies for Biomedicine, Engelhardt Institute of Molecular Biology, Russian Academy of Sciences, 119991 Moscow, Russia

**Keywords:** teratocarcinoma, human endogenous retroviruses, proteins, deglycosylation, electron microscopy, viral RNA, RT-PCR, cloning, microvesicles

## Abstract

About 8% of our genome is composed of sequences from Human Endogenous Retroviruses (HERVs). The HERV-K (HML.2) family, here abbreviated HML.2, is able to produce virus particles that were detected in cell lines, malignant tumors and in autoimmune diseases. Parameters and properties of HML.2 released from teratocarcinoma cell lines GH and Tera-1 were investigated in detail. In most experiments, analyzed viruses were purified by density gradient centrifugation. HML.2 structural proteins, reverse transcriptase (RT) activity, viral RNA (vRNA) and particle morphology were analyzed. The HML.2 markers were predominantly detected in fractions with a buoyant density of 1.16 g/cm^3^. Deglycosylation of TM revealed truncated forms of transmembrane (TM) protein. Free virions and extracellular vesicles (presumably microvesicles—MVs) with HML.2 elements, including budding intermediates, were detected by electron microscopy. Viral elements and assembled virions captured and exported by MVs can boost specific immune responses and trigger immunomodulation in recipient cells. Sequencing of cDNA clones demonstrated exclusive presence of HERV-K108 *env* in HML.2 from Tera-1 cells. Not counting two recombinant variants, four known *env* sequences were found in HML.2 from GH cells. Obtained results shed light on parameters and morphology of HML.2. A possible mechanism of HML.2-induced diseases is discussed.

## 1. Introduction

Endogenous retroviruses (ERVs) are fossils of exogenous retroviruses that initially integrated into the DNA of a germinal cell and by that distributed over the body millions of years ago [1,2]. About 8% of the human genome is composed of sequences derived from human ERVs (HERVs), while the amount of protein coding genes in human genome is below 2% [3]. Most endogenous retroviruses are silent because of epigenetic repression and post-insertional deletions or insertions.

An HERV-K (HML.2) family is recently integrated and the best presented viral family of endogenous betaretroviruses, here abbreviated as HML.2. About 100 of them are presenting partial or full-length genomes with open reading frames [4]. They are distantly related to the Mouse Mammary Tumor Virus (MMTV) and the Jaagsiekte Sheep Retrovirus (JSRV). The amino acid identity between HML.2 Gag and Env proteins and corresponding proteins of exogenous retroviruses (MMTV, JSRV) are not exceeding 30%. Another demonstration of distant relationship of HML.2 and exogenous betaretroviruses is glycosylation rate. Dependent on the HML.2 variant and, excluding the signal peptide, there are 11 N-glycosylation sites in Env, including four sites in transmembrane (TM) protein, while most exogenous betaretroviruses are less glycosylated (Table 1). Finally, formation of the hexametric Gag lattice of HML.2 occurs at the plasma membrane, while capsids of exogenous betaretroviruses are assembled in the cytoplasm [5].

Despite defectiveness in at least one gene, several HML.2 are coding for functional proteins [4]. In particular, this includes the Env protein of HERV-K108b [6]. Interestingly, not all proviral loci are present in the gnome of every person [7]. For example, HML.2 with complete genomes—HERV-K113 and HERV-K115—are polymorphic, and they were detected in approximately 30% and 15% of the human population, accordingly [8]. HML.2 loci are also categorized by type. Proviruses with a characteristic 292 bp deletion at the N-terminal part in *pol-env* junction are referred to as type 1, while the regular loci without this deletion are type 2. The deletion leads to alternative splicing and, as a consequence, type 1 elements are incapable of coding for Env or Rec proteins [9]. However, they express an accessory protein called “Np9”, known to down regulate nuclear antigen 2 of Epstein–Barr virus [10]. Np9 is also engaged in the regulation of the p53-MDM2 pathway in humans and primates [11].

HERV expression, including that of HML.2, was noted in malignant tumors such as human breast cancer, ovarian cancer, germinal tumors, melanomas [12,13,14,15,16,17] and in association with multiple sclerosis, and other autoimmune and neurological disorders [18,19,20,21,22]. Implication in triggering cancer and neurological diseases is controversially debated [23,24]. It is still unclear whether viruses are inducers of cell transformation, or whether expression is triggered by cell deregulation (e.g., promoter demethylation) during carcinogenesis. HML.2 can be activated by exogenous viruses such as Human T-cell Leukemia Virus (HTLV-1) and HIV-1 [25,26,27]. As a consequence of a low virus titer, parameters and properties of the wild type HML.2 from malignant tumors were not investigated in detail. This problem was partially solved by establishing several human cell lines including teratocarcinoma cell lines GH and Tera-1 [28] expressing HML.2. Viruses from these cell lines further abbreviated as HML.2 GH and HML.2 Tera-1.

To obtain sufficient amount of HML.2 viruses from teratocarcinoma cell lines, tens of liters of cell supernatant were harvested and processed [14,29]. An alternative for raising particle production was stimulation of cells with hormones, and nucleoside analogs, like 5-bromodeoxyuridine, or 5-iododeoxyuridine, but this might lead to aberrant expression with consequences for particle formation [14,29]. Attempts to infect human and animal indicator cells by co-cultivation with HML.2 from teratocarcinoma cell lines failed [14]. Earlier, to visualize virions and study morphology, transmission electron microscopy (TEM) of HML.2 particles was applied [29,30,31]. Only bald virions with immature doughnut-like capsids were seen, while mature virions with collapsed (or condensed) capsids and surface projections (SP) were not detected [31].

As a consequence of low HML.2 expression, several additional approaches were developed. In particular, transfection of cells with expression vector containing HML.2 genes under strong promoters and utilization of constructs with codon optimized sequences [32]. Using a full-length molecular clone, the HML.2 particles were successfully generated in baculoviruses [33]. HML.2 elements were also studied separately. For example, Gag was studied using expressing recombinant poxviruses and Env function has been analyzed by pseudo-types composed of capsids from lentiviruses [34,35]. These methodologies allow producing significant amounts of virus-like particles for research. However, a massive transfection of cells with a set of expression vectors might lead to overproduction of viral proteins and enzymes. As cell machinery has a certain limit of protein processing and modifications [36], overproduction of recombinant proteins can hump these processes and disturb assembly of virions.

Here, results of comprehensive analyses of HML.2 from not stimulated human teratocarcinoma cell lines are presented. To isolate viruses’ cells, supernatants were concentrated 2000 times. To reduce the amount of debris, most studies were performed on viruses purified by sucrose density gradient centrifugation (Gdc). The implication of presumed microvesicles (MVs) in a hijack of HML.2 elements during particles assembly on plasma membrane was noted. The viral cargo delivered by MVs can induce prominent immunomodulation in recipient cells. Obtained results extend our knowledge of HML.2 and describe events that might lead to diseases.

## 2. Results

### 2.1. Proteins and Parameters of HML.2 from Teratocarcinoma Cell Lines GH and Tera-1

**Figure 1:**
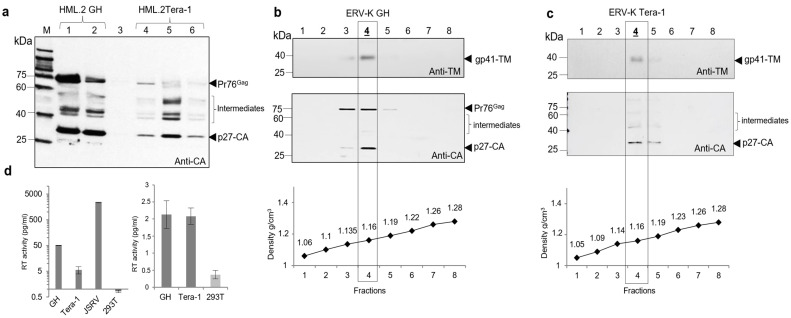
Protein pattern of HML.2 GH and HML.2 Tera-1, buoyant density of virions and viral RT activity. (**a**) Viruses were purified on 20% sucrose cushion and concentrated (conc. × 2K). Detection of Pr76^Gag^, intermediate Gag cleavage products and p27-CA in HML.2 GH and HML.2 Tera-1 by Western blot analysis. Tracks 1, 2—HML.2 GH; Tracks 4–6—HML.2 Tera-1. Track 3—Supernatant from HEK293T*. M1—Size markers (VisiBlot, Serva, Heidelberg, Germany). Western blot analysis was performed using rabbit anti-p27-CA serum. Seven liters of each sample were loaded on the gel. *Supernatant from HEK293T cells was harvested after four days in culture, processed like virus containing supernatant (conc. × 2K); (**b**) detection of TM (upper panel) and CA (lower panel) of HML.2 GH in fractions of the gradient after centrifugation. Western blot analyses were performed using anti-TM Mab (upper panel) and rabbit anti-p27-CA serum (lower panel). Tracks 1–8 correspond to subsequent fractions of the gradient; (**c**) same analysis as above but testing HML.2 Tera-1. M—Size markers (VisiBlot, Serva, Heidelberg, Germany). Buoyant density of gradient fractions is given below the figures. Fractions F4 (1.16 g/cm^3^) in which the highest amount of viral proteins were found are shown in frame. (**d**) The RT activity (pg/mL) was measured in HML.2 GH and HML.2 Tera-1 (conc. × 2K) purified on sucrose cushion (left panel). JSRV (conc. × 0.5K) and HEK293T (conc. × 2K), were used as positive and negative controls, respectively. Then, HML.2 GH and Tera-1 from F4 (1.16 g/cm^3^) were tested (right panel). RT activity was low (~2 pg/mL), and it was nearly equal in tested samples. In both experiments, five liters of each sample were examined. *Supernatant from 293 T cells was harvested after four days in culture and processed and concentrated (conc. × 2K), like virus containing supernatants.

The initial aim of this study was to characterize virus proteins, estimate buoyant density of viruses and measure the RT activity of HML.2 GH and HML.2 Tera-1. Before starting the work, a protocol of virus isolation was optimized. In order to prevent potential formation of aberrant particles by overexpression of viral proteins, GH and Tera-1 cells were grown in regular growth medium without additional hormones or other stimulants. To diminish possible destruction of virions by freeze-thawing procedure, cell supernatants from GH and Tera-1 cells were processed the same day including supernatant centrifugation, filtration and concentration through the 20% sucrose cushion, as described (Section 4.3). Obtained virus pellets were diluted to achieve 2000 times concentrates and analyzed by Western blot analyses using anti-p27-CA serum (Figure 1a). It was shown that the amount of Gag proteins was 4–5 times higher (estimated using ImageJ) in concentrated supernatants of GH cells, compared to that of Tera-1 cells. A significant amount of HML.2 GH contained unprocessed precursors Pr76^Gag^ denote impaired maturation of capsids, while there were less unprocessed Pr76^Gag^ in HML.2 Tera-1 preparations. Intermediate cleavage products of 50–42 kDa were detected in both preparations, indicating circulation of certain amounts of virions with incompletely assembled capsids (Figure 1a).

Subsequently, we used sucrose density gradient centrifugation (Dgc) to estimate the viral buoyant density and distribution of proteins. Cell supernatants were prepared as described (Section 4.3). After Dgc, fractions were harvested, diluted in TN buffer, pelleted again and analyzed by Western blot analysis (Figure 1b,c). The highest concentration of CA and TM proteins in both gradients was detected in fractions number 4 (F4) with a buoyant density of 1.16 g/cm^3^ (in frame). However, “light” content mostly composed of Pr76^Gag^ was noted in F3 (1.135 g/cm^3^) (Figure 1b). Reduced density might be a consequence of HML.2 GH interaction with “light” extracellular vesicles or a result of low vRNA content.

### 2.2. Reverse Transcriptase (RT) Activity in HML.2 GH and Tera-1

The HML.2-associated RT activity was estimated using a sensitive product-enhanced RT (PERT) assay. Cell supernatants were harvested after 72 h and processed as described (Section 4.3). The first assay was performed using HML.2 purified on a sucrose cushion. Three preparation of HML.2 GH and two preparations of HML.2 Tera-1 (conc. × 2K) were tested. Five microliters of each sample (equal to 10 mL of supernatant) were used per reaction. The RT activity of HML.2 GH was estimated as 50 pg/mL, while, in HML.2 Tera-1, it was ~5 pg/mL (Figure 1d, left panel). However, as shown before (Figure 1a), the amount of HML.2 Tera-1 Gag proteins were 4–5 times lower than that of HML.2 GH. Thus, if adjusting the CA contents, the RT activity, compared to that of HML.2 GH, would be two times lower. JSRV purified on sucrose cushion (conc. × 0.5 K) was as a positive control and supernatant from HEK293T cells (conc. × 2 K) and used as a negative control. The second assay was performed using five microliters of pelleted and diluted viruses from gradient fractions (1.16 g/cm^3^). A disclosed RT amount in HML.2 was low, but it was nearly equal (~2 pg/mL) in compared samples (Figure 1d, right panel). As ERV-K Tera-1 has a lower Gag content (shown above), revealed equal RT activity in viruses indicated that Tera-1 cells might discharge more particles with RT.

### 2.3. Deglycosylation of HML.2 GH and HML.2 Tera-1

Deglycosylation discloses two basic parameters of glycoproteins: molecular mass of the backbone protein and total amount of associated glycans. Previously deglycosylation studies on HML.2 Env were performed using recombinant viruses [6,35]. For the first time, deglycosylation studies were performed on HML.2 from cell lines.

The HML.2 Env proteins devoid of the signal peptide contain 11 N-glycosylation sites; seven are located in SU and four in TM [35,37,38] (Table 1). Whether the sites are glycosylated and what kind of glycans are attached depends largely on the cell type and glycosyltransferases repertoire. The molecular mass of attached carbohydrates might vary from ~2.5 kDa in bi-antennary types to ~5 kDa in penta-antennary complexes.

**Figure 2:**
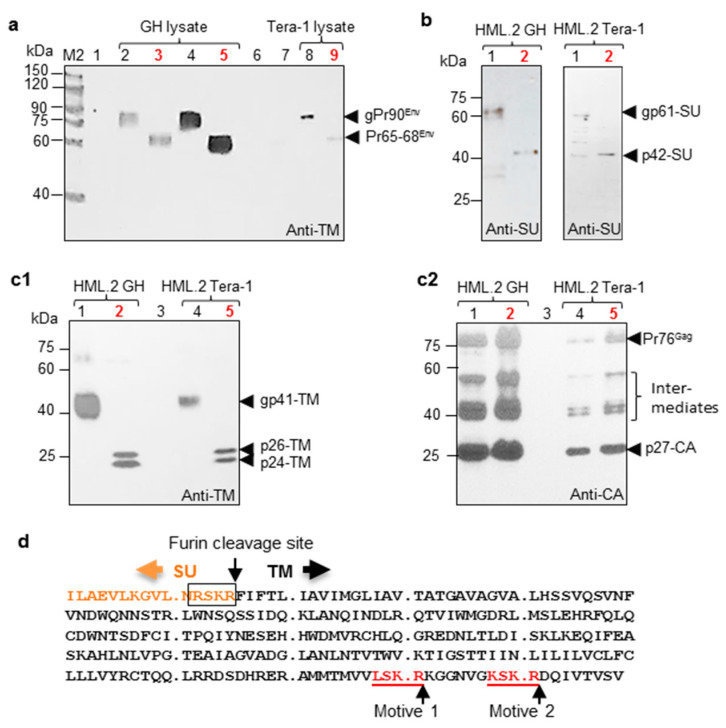
Deglycosylation of HML.2 Env precursors and HML.2 GH and Tera-1 purified on 20% sucrose cushion. Estimation of SU and TM glycosylation and detection of truncated TM variant. (**a**) cellular lysates treated with PNGase F. Treated samples are marked in red. M2—Size markers. Tracks 1, 6 and 7—Lysates of HEK293T cells (negative controls); Tracks 2 and 4—Non-treated lysate of GH cells; Tracks 3 and 5—Treated GH lysates; Track 8—Non-treated lysate of Tera-1 cells; Track 9—treated lysate. Western blotting was performed with anti-TM Mab. Positions of Env precursors and deglycosylated backbones are indicated by arrow heads. (**b**) Left panel—Treatment of HML.2 GH SU. Track 1—Non-treated; Track 2—Treated. Right panel—treatment of HML.2 Tera-1 SU. Track 1—Non-treated; Track 2—Treated. Positions of gp61-SU and backbone p42-SU are marked with arrowheads. Western blot analysis was performed using rabbit anti-SU serum. (**c1**) Treatment of HML.2 GH and Tera-1 purified on sucrose cushion. Tracks 1—Non-treated HML.2 GH; Track 2—treated HML.2 GH; Track 3—Negative control (Supernatant from 293T cells processed like virus containing supernatant (conc. × 2K)); Tracks 4 and 5—Non-treated and treated HML.2 Tera-1, respectively. In addition to TM a truncated TM (p24-TM) was revealed after deglycosylation of gp41TM. Truncation likely occurs at the C-terminus of TM. Blot was incubated with anti-TM Mab. (**c2**) To confirm integrity of analyzed HML.2, the membrane (**c1**) was stripped and re-probed with anti-p27-CA serum. The positions of Gag precursor Pr76^Gag^ and p27-CA are marked; (**d**) the SU-TM cleavage site (in frame) and two presumed furin cleavage motives (red, underlined) at the C-terminus of TM. Sequence integrity and position of putative motives are demonstrated on one of the cloned sequences.

First, GH and Tera-1 total cell lysates were analyzed for Env precursors by treatment with PNGase F. Total cell lysates may contain different variants of precursors, including those with or without signal peptides and precursors at different stages of glycosylation. As a consequence of that, protein bands in Western blot analyses may appear as fuzzy, while the presence of incompletely glycosylated forms can reduce the molecular mass of analyzed molecules. As shown by Western blot analysis, in non-treated cellular lysates of GH cells, Env precursor(s) appeared as a “diffused” band of about 90 kDa (Figure 2a). Deglycosylation of the precursors resulted in a backbone of ~68 kDa (Figure 2a), while diffused bands observed after deglycosylation are likely reflecting some diversification of HML.2 GH genes expressing Env. A sharper band was detected in Tera-1 cell lysate, indicating the identity of expressing Env, while the amount of precursors in Tera-1 cells, compared to GH cells, was significantly lower (Figure 2a). The difference between treated and non-treated precursors from cell lines was 20–23 kDa.

Next, we performed deglycosylation of gp61-SU from HML.2 GH purified on sucrose cushion. After deglycosylation, a backbone of 42 kDa (p42-SU) was detected, which had the expected theoretical mass according to the consensus sequence [37,38]. This treatment reduced molecular mass of the protein by ~18 kDa (Figure 2b left). If count bi-antennary glycans, it means that all seven sites in SU might be deglycosylation. The same procedure was performed on HML.2 Tera-1 and obtained results were similar, but signals were weaker (Figure 2b right). It was shown that glycosylated TM has a molecular mass of 41 kDa and PNGase F treatment caused ~16 kDa mobility shift (Figure 2(c1)). Interestingly, after PNGase treatment of TM, two separate proteins with molecular mass of 26 kDa (p26) and 24 kDa (p24) appeared (Figure 2(c1)). The membrane was stripped and re-probed with anti-p27-CA to confirm integrity of analyzed viruses (Figure 2(c2)). To explain the origin of p24-TM, we reasoned that a deletion in TM or a premature stop-codon might result in a shorter TM in a significant proportion of particles. To check these possibilities, the viral RNA from fractions F4 (1.16 g/cm^3^) was reverse transcribed. Two regions covering the membrane spanning part and the intracellular part of TM were amplified by PCR using the primer pairs 5′TM-LTR and 3′SU-LTR. RT-PCR of amplicons revealed in each case only one band of the expected size (468 bp or 940 bp, respectively). The 940 bp amplicons from both cell lines were cloned, and 10 clones were sequenced. Sequencing did not show deletions or premature termination in TM, suggesting that truncation of TM occurs at a post-translation level. Further on, in silico analysis of putative furin-like cleavage site (PropP 1.0 server) revealed two motives that contained three out of four amino acids, which match the SU-TM furin cleavage site (Figure 2d). However, these motives were estimated as “not strong”. However, if cleavage occurs, that would likely be after motive #1 (LSKR), which was ranked slightly higher than motive #2. Motive #1 is located 18 amino acids (=1.87 kDa) away from the C-terminus. The difference of ~2 kDa between p26-TM and truncated p24-TM fits well with a shift revealed by Western blot analyses (Figure 2(c1)). No motives resembling furin cleavage site were detected at the N-terminal part of TM.

Altogether, the glycosylation moieties of HML.2 GH Env contribute to ~32 kDa. This is approximately 30% of a total molecular mass of the Env precursor. Depending on the complexity of glycans, about 9–11 sites might be glycosylation. 

### 2.4. Analyses of Viral Particles and Extracellular Vesicles by Negative Staining and Ultrathin Section TEM

**Figure 3:**
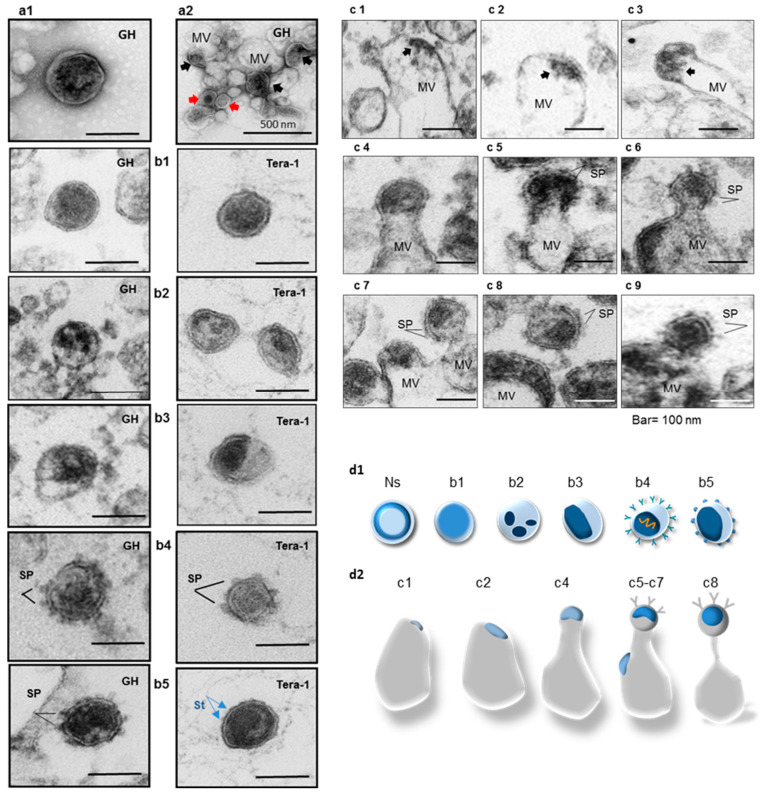
Morphology of HML.2 GH and HML.2 Tera-1 isolated from sucrose gradient fractions with a buoyant density 1.16 g/cm^3^. Demonstration of viral elements capture by presumed MVs. Particles were examined by negative staining (**a**) and by ultrathin section (**b**,**c**) TEM. Cartoons of capsids and envelopes of the virus-like particles (**d1**) and MVs with captured viral cargo (**d2**). (**a1**) Negative staining. Bald particles with amorphous capsids were detected in HML.2 GH preparation. (**a2**) HML.2 GH preparation that was not filtered through the 0.45 µm membrane but purified by sucrose Dgc. Obtained pellets were diluted and analyzed. Aggregate composed of extracellular vesicles with attached virus-like particles (red arrows) and dense virus-like content inside vesicles are shown (black arrows); (**b**) comparative analyses of HML.2 GH and HML.2 Tera-1 free virions; (**b1**) bald virion with amorphous capsid; (**b2**) bald virion with fragmented capsids; (**b3**) bald virions with condensed ovoid eccentric capsids; (**b4**) virions with SP and partially condensed capsids; (**b5**) virion with SP (left) and virion with partially condensed capsid with presumed TM stumps (St, blue arrows) (right); (**c**) capture of viral elements (black arrows) at different steps of assembly by presumed MVs; (**c1**,**c2**) supposed Gag complexes under the cell membrane (early budding stage); (**c3**–**c5**) assembly of viral capsid (mid budding stage); (**c5**) SP at the surface of particle in formation; (**c6**) assembled particle, constriction of the neck before completion of the budding (late budding stage); (**c7**) three virions at different stages of assembly trapped inside overlapped MVs. Particle (upper right) with constriction neck, condensed capsid and SP (late budding stage); (**c8**) assembled virion with SP attached to MV; (**c9**) supposed free virion with SP and amorphous capsid. Bars represent 100 nm. MV: Microvesicle. SP: spike proteins (or surface projections) at the surface; (**d1**) graphical interpretation of obtained results. Morphology of HML.2 free particles from teratocarcinoma cells (upper panel). Immature particles with toroidal capsids are not shown (Ns). Particle with amorphous capsid is shown in light blue and condensed capsids are given in dark blue; (**d2**) variants of HML.2 viral cargo in MVs. Virions with SP (shown as “Y”) and condensed capsids were not frequent. Virions with vRNA (shown as a “spring”) are not frequent.

Initially, viruses from the main fractions (1.16 g/cm^3^) of HML.2 GH and Tera-1 were analyzed by negative staining. This is a relatively simple and quick method for virus detection, while, in the presence of extracellular vesicles, retrovirus particles are more difficult to differentiate because internal structures frequently remain invisible. Despite thorough analyses of the fields, virus-like structures with morphology of betaretroviruses were not clearly identified, whereas, several bald particles of 100–120 nm in diameter with amorphous capsids were detected (Figure 3(a1)). We then attempted to detect extracellular vesicles that might be present in supernatant together with HML.2. To fully appreciate vesicles of different sizes, filtration of supernatant through the 0.45 µm membrane filter was omitted, while ultra-centrifugation through a sucrose cushion and Dgc were performed. Vesicles of 70 nm to 300 nm in diameter and aggregates of vesicles with “attached” bald virus-like particles of ~100 nm in diameter were found (red arrowheads) (Figure 3(a2)). In several vesicles, dense content (black arrowheads), presumably of viral origin, was present. It should be noted that vesicles above 100 nm, but less than 1000 nm in diameter, are microvesicles [39]. Thus, it was demonstrated that HML.2 GH preparation contained a significant amount of extracellular vesicles. 

Internal structures of HML.2 particles were analyzed by ultrathin section TEM. A significant amount of vesicles and virus-like particles with diameter 110 nm ± 20 nm (*n* = 50) were detected in the examined field. Most of the viral capsids were of irregular shape. Particles with amorphous capsids of ~80 nm in diameter with no lucent space between the capsid and the envelope were predominant (Figure 3(b1)). Free particles with partially condensed or condensed (or collapsed) capsids were less frequent. The mean diameter of the capsids was 50 ± 20 nm. Certain capsids have an ellipsoid shape (Figure 3(b2) Tera-1, Figure 3(b3) Tera-1). Several free particles contained 6–8 nm long SP but did not appear to depend on the capsid morphology. A significant amount of HML.2 elements were found inside presumed MVs (Figure 3(c1–c9)), and budding structures were occasionally seen (Figure 3(c6–c9)). A brief description of HML.2 GH and HML.2 Tera-1 morphology is given below:

Group 1—Bald particles with amorphous capsids (Figure 3(b1)). About 60–70%.
Group 2—Bald particles with fragmented capsids (Figure 3(b2)). Not frequent.
Group 3—Bald particles with condensed (Figure 3(b3)) or partially (incompletely) condensed capsids (65–80 nm in diameter), with partial lucent space between the capsid and the envelope (Figure 3(b3)). About 10–15%.
Group 4—Particles with 6–8 nm long SP and condensed or partially condensed capsids about 65–80 nm in diameter (Figure 3(b4,b5) left). Such particles were not frequent (~1%). Several virions with partially condensed capsids and small surface protrusions (~2–3 nm) located at nearly equal distance between each other were detected. These protrusions are likely representing the TM stumps (Figure 3(b5) right).
Group 5—Extracellular vesicles with captured “viral elements” displayed a variety of vesicular assemblies containing HML.2 GH budding structures with and without SP (Figure 3(c1–c9)). Association of HML.2 Tera-1 with MVs was exceptionally rare.

Lastly, only few particles with immature doughnut-like (toroidal) capsids were detected (not shown). A low amount of such particles indicated that most of the particles passed the initial steps of capsid maturation. Graphical interpretation of observed free virus particles is shown (Figure 3(d1)).

### 2.5. Search for Virus Diversification in HML.2 Pool and Analyses of SU and vRNA in Fractions of the Sucrose Density Gradient

Diversity of shapes and variable morphology of HML.2 capsids suggested that viruses with different parameters and properties might be released from these cells. Previously, diversification of HTLV-1 was shown in human lymphoid cell line MT-2 [40].

**Figure 4:**
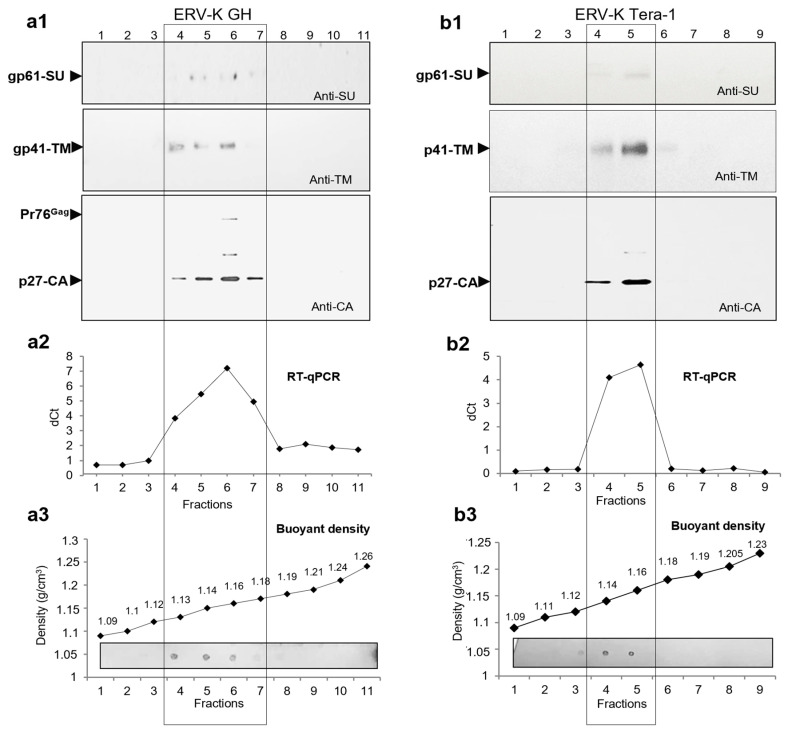
Searching for diversification in HML.2 GH pool and analyses of SU, TM and vRNA in fractions of the gradient. (**a1**) analyses of HML.2 GH using extended 20–60% sucrose gradient. Viral SU, MA and CA proteins were detected by Western blot analyses; (**a2**) viral RNA in fractions of the gradient was analyzed by RT-qPCR. A minor hump seen on the cDNA curve F4 (1.13 g/cm^3^) most likely represented fragmented virus elements in association with “light” MVs, but not a separate population of assembled viruses; (**a3**) buoyant density of gradient fractions and total protein content in fractions using dot blot (lower panel) are shown; (**b1**) analyses of HML.2 Tera-1 were performed in a similar way, but using a standard 20–60% sucrose gradient. Signs of the virus’s diversification were not detected. A low amount of SU was shown in both preparations; (**b2**) viral RNA distribution along the gradient; (**b3**) buoyant density of gradient fractions and total protein content in fractions of the gradient estimated by a dot blot (low panel). Western blot analyses were performed using rabbit anti-SU serum (upper panel), mouse anti-TM Mab (middle panel) and rabbit anti-CA (low panel). Viral RNA content was examined by RT-qPCR. Delta threshold cycle (Ct) values were obtained by subtraction of RT-qPCR Ct values from Ct values did during qPCR without RT step. Virus-specific proteins are marked with arrow heads. Fractions with the highest protein and vRNA contents are in frame.

Thus, to accomplish characterization of HML.2 from teratocarcinoma cell lines, analyses of viruses diversification, SU and vRNA, that were not previously performed, were carried out. As shown by Western blot analyses (Figure 1b), gradient fraction F3 (1135 g/cm^3^) contained Pr76^Gag^ and traces of CA and TM. Considering this result and disclosed diversity of viral particles from GH cells, it was suggested that virus diversification might take place in the HML.2 GH pool. To answer the question, sucrose gradient was modified. For better separation within the “viral-layer” (1.13–1.18 g/cm^3^), an additional 30% sucrose layer was added between 20% and 40% layers (Figure 4(a1)). After sucrose Dgc (Section 4.3), eleven fractions of the gradient were examined for protein content by Western blot analysis and for vRNA by RT-qPCR, targeting the gag gene.

Viral proteins were detected by Western blot analysis in fractions F4–F7 with buoyant densities 1.134–1.185 g/cm^3^ (Figure 4(a1)). As the CA and vRNA contents were gradually increasing from F4 to F6, the presence of a separate type of particles was unlikely. A minor hump (Figure 4(a2)) seen on the cDNA curve might be formed by MVs of a low density and a large diameter, which might contain partially assembled capsids with vRNA. A combination of a high amount of TM, low amounts of CA and vRNA in F4 likely indicated the presence of MVs with hijacked HML.2 elements. Total protein dot blot analyses demonstrated the highest content in F4 (Figure 4(a3) bottom). However, as shown by Western blot analysis, this fraction contained the lowest amount of CA (Figure 4(a1) bottom). The highest viral protein and vRNA contents were detected in F6 (buoyant density 1.155 g/cm^3^) (Figure 4(a1,a2)). Analyses of HML.2 Tera-1 revealed proteins and vRNA in two fractions of the gradient F4-F5, thus demonstrating homogeneity of the virus pool (Figure 4(b2)). Detection of the HML.2 SU was problematic. Traces of the protein were found in F5-F6 of HML.2 GH and in F5 of HML.2 Tera-1 (Figure 4(b1)). These data confirmed results obtained by TEM, which postulated an insignificant amount of HML.2 particles with SP. A good co-localization of HML.2 proteins with vRNA in fractions of the gradient confirmed the integrity of analyzed viruses.

### 2.6. Viral RNA Packaged in HML.2 GH and HML.2 Tera-1, Analyses of env Type 2 Gene

**Figure 5:**
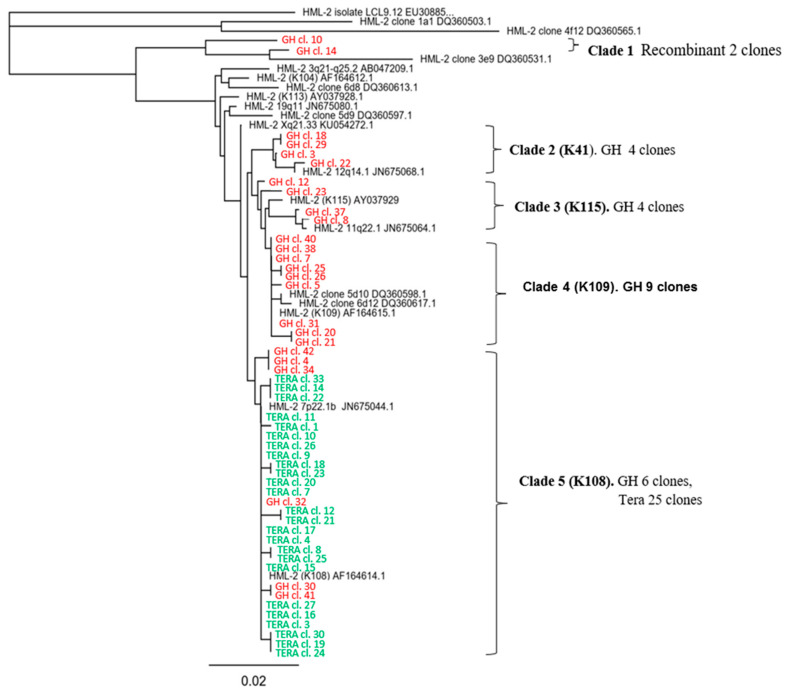
Genetic diversity of transcripts packaged in HML.2 GH and HML.2 Tera-1 particles. Analyses of type 2 *env* genes. Neighbor-joining phylogenetic analysis of 460 bp *env* cloned sequences. Viral RNA was extracted from gradient fractions with buoyant density 1.16 g/cm^3^. Twenty-five sequences from HML.2 GH (red) and from HML.2 Tera-1 (green), and nineteen reference sequences (black) were used for alignment. Cloned sequences formed five clades. HML.2 Tera-1 contained HERV-K 108 *env* and closely related sequences (Clade 5), while HML.2 GH packaged diverse *env* (Clades 1–5).

Viral RNA from fraction F4 (Figure 1a,b) was RT-PCR amplified using an HML.2 *env* type 2 specific sense primer and a generic antisense primer. Amplicons of 460 bp were cloned and sequenced. In total, 25 clones from each virus were sequenced and aligned to 19 HML.2 reference sequences. A neighbor-joining phylogenetic analysis of 69 sequences is presented (Figure 5). Sequencing of HML.2 Tera-1 *env* clones revealed that 25/25 clones (Clade 5) formed a cluster with HERV-K108 (7p22.1b) *env*. Twelve clones were identical with the reference sequence, while the remaining 13 clones demonstrated predominantly a single nucleotide polymorphism, while reading frames were not changed. Cloning and sequencing of HML.2 GH demonstrated a diversity of *env* sequences. Nine clones formed a cluster with HERV-K109 (Clade 4) and six clones clustered with HERV-K108 (Clade 5). Four clones were close to HERV-K41 (Clade 2) and to HERV-K115 (Clade 3), respectively. Two related recombinant sequences that contained fragments of HERV-K108 *env* and HERV-K109 *env* were found (Clade 1).

### 2.7. Quantification of HML.2 Particles Released from GH and Tera-1 Cells

It is well established that the number of HML.2 particles released from teratocarcinoma cells is low, but how low it is was not estimated. We intended to quantify viral particles to estimate production of HML.2 by teratocarcinoma cell lines and extrapolate the data to possible viral circulation in vivo. The quantification was based on the HML.2 CA content detected by Western blot analyses using a concentrated cell free supernatant. The quantification also included CA molecules that might be present in vesicular entities containing virus-like budding structures, as observed by TEM (Figure 2b).

**Figure 6:**
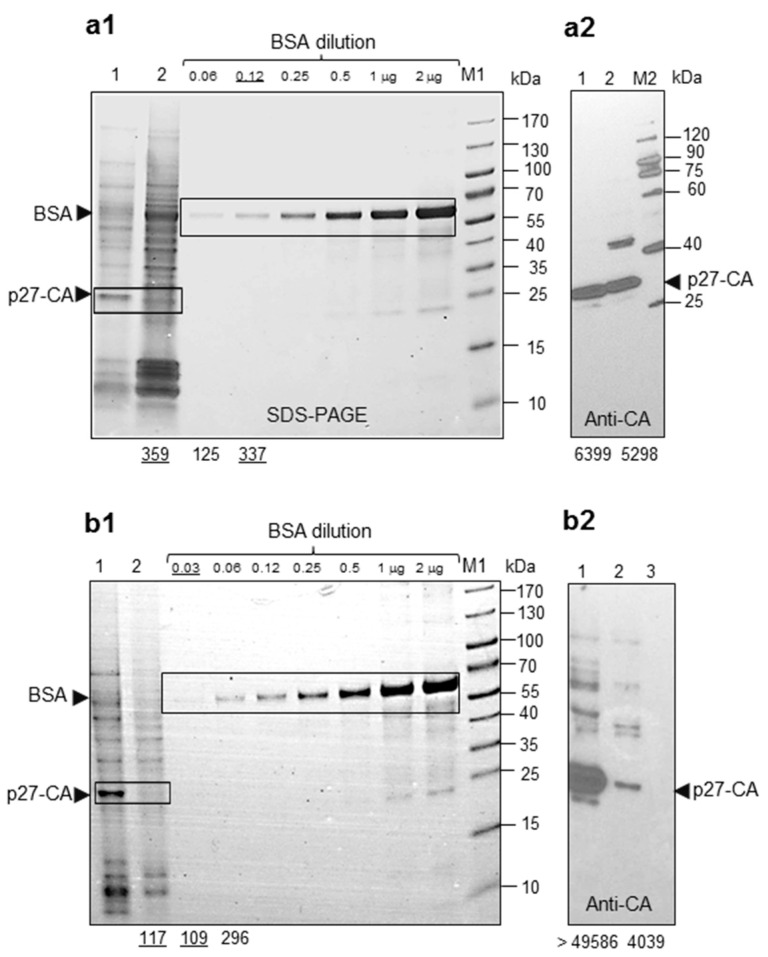
Estimation of CA/virions release from GH (**a**) and Tera-1 cells (**b**). Viruses were purified on 20% sucrose cushion and concentrated × 2 K, as described (Section 4.3). Serial dilution of bovine serum albumin (BSA, Fraction V) was used for protein quantification. BSA load (ng/µg) is indicated above the tracks. (**a1**) estimation of CA release from GH cells. Track 1. Recombinant HML.2 (conc. × 200) used for direct CA comparison and comparative Western blot analyses, load 8 µL; Track 2—HML.2 GH, load 8 µL. After electrophoresis the gradient gel was stained with Fast-blue (Roth, Germany). (**a2**) comparison of CA amount in recombinant ERV-K and in ERV-K GH by Western blot analysis using anti-p27-CA serum; (**b1**) estimation of HML.2 CA released from Tera-1 cells. The same methodology as above (a1) was applied; (**b2**) track 1. Recombinant HML.2 (conc. × 200), load 8 µL; Track 2. HML.2 Tera-1, load 8 µL; Track 3. Supernatant from HEK293T cells (conc. × 2 K). Size markers M1 (PageRuler) and M2 (VisiBlot) were used for calibration. A comparison of protein load was performed using ImageJ. Numbers below the figures are corresponding to the relative intensity of the bands. Underlined numbers indicated close intensity of the reference and examined sample bands.

Cell supernatants were harvested after four days in culture and processed as described (Section 4.3). Viral pellets after centrifugation through 20% sucrose were diluted to obtain × 2K concentrates. Serial dilutions of bovine serum albumin (BSA) were used as references for quantification of band intensity after SDS-PAGE gel staining. Recombinant HML.2 particles produced by transfection of HEK 293T cells with expression vectors (Section 4.4) were used as a control of CA content by Western blot analyses. After electrophoresis, one part of the gel was stained with Fast-blue (Figure 6(a1)) and another with the same amount of HML.2 GH was used for comparative Western blot analysis (Figure 6(a2)). Staining of the gel demonstrated that the intensity of 120 ng band of BSA corresponded to CA band of HML.2 GH (Figure 6(a1)). A close to equal amount of CA in recombinant HML.2 and in HML.2 GH was shown by Western blotting using anti-p27-CA serum (Figure 6(a2)). The number of CA proteins in the assembled HML.2 capsid was presumed to be close to that in HIV-1 capsid, which is about 2100 CA molecules or 0.1 fg [41,42]. As 16 mL of cell supernatant contain about 120 ng of CA, there are 7.5 ng of CA molecules per mL. One nanogram of CA contains 2.1 × 10^10^ molecules. There are theoretically 1.5 × 10^11^ CA molecules or 7.5 × 10^7^ particles in one mL of GH cell free supernatant harvested after four days. This corresponds to 4 × 10^10^ CA molecules or 1.8 × 10^7^ particles per day.

Next, we attempted to quantify the CA molecules which might be released from one GH cell and one Tera-1 cell. Cells were growing in T175 flask in 35 mL of culture medium and there were 2.3 × 10^7^ cells at confluence. Thus, 1 mL of culture medium might contain a “discharge” from 6.5 × 10^5^ cells. Dividing 1.5 × 10^11^ CA molecules by 6.5 × 10^5^ cells results in 2.3 × 10^5^ CA molecules produced by a single cell after four days. Thus, without taking decay (particle/CA) into account, the mean production rate of a single GH cell is about 56,700 CA molecules, or 27 virions per day. These numbers represent the highest possible amount of CA production. As shown by TEM, a significant number of CA molecules at different stages of assembly are trapped inside MVs, thus the amount of free virions might be significantly lower.

Similar estimations were performed on HML.2 from Tera-1 cells (Figure 6b). As after gel staining the CA band was rather weak, estimation error might be more prominent. The CA band intensity was close to the 30 ng band of BSA. Thus, 1 mL of supernatant from Tera-1 cells contained 1.9 ng of CA. An analogous calculation as that above indicated that ~6 capsids/virions might be released per day from a single Tera-1 cell.

## 3. Discussion

A low level of virus production is one of the principal obstacles in HML.2 structural and functional research. This is why, despite intense studies performed previously, several parameters and properties of these viruses were not investigated in detail. Here we present results of comprehensive analyses of HML.2 from teratocarcinoma cell lines GH and Tera-1.

The first part of the study was focused on viral proteins, buoyant density of virions and viral RT activity. Detailed examination of HML.2 Tera-1 protein pattern and vRNA content was performed for the first time, as previous analysis of HML.2 Tera-1 revealed only Gag precursor [31]. HML.2 GH and HML.2 Tera-1 purified on a sucrose cushion were evaluated by Western blot analysis. Judging by the CA content, the amount of HML.2 GH was 4–5 times higher, compared to the HML.2 Tera-1. It was proposed that this difference was a result of active expression of several HML.2 loci in GH cells and that was confirmed by analyses of vRNA (Section 2.5). Western blot analyses of gradient fractions showed that both CA and TM proteins were predominantly associated with fractions with buoyant densities of 1.16 g/cm^3^, typical for retroviruses.

Next, the HML.2-associated RT activity was examined using PERT assay. It was shown that the RT activity compared to exogenous retrovirus was low (Figure 1d). We attempt to estimate putative amount of HML.2 particles with RT, knowing that 50–100 molecules of RT per virion are required for reverse transcription [43]. The molecular mass of monomeric RT of MMTV and HML.2 is 66 kDa [44,45]. Five µL of virus preparation after centrifugation through sucrose cushion were concentrated from 10 mL of GH cell supernatant. Detected 50 pg of RT corresponds to 5 × 10^8^ molecules. Thus, one mL contained 5 × 10^7^ RT molecules. If counting 100 molecules of RT/virion, it gives 5 × 10^5^ virions with RT. Our estimation (Section 2.7) indicated that 7.5 × 10^7^ cores/virions might be present in one mL of supernatant from GH cells. Dividing total virion content by the number of virions with RT would give 1/150 in case of HML.2 GH and 1/300 in case HML.2 Tera-1 particles with RT, respectively. However, RT results were different when viruses purified by sucrose Dgc were analyzed. A similar RT activity was revealed in viral samples. As the HML.2 Tera-1 contained 4–5 times less viruses compared to HML.2 GH, the amount of RT in HML.2 Tera-1 is likely higher. This might be a consequence of a better Gag-Pol cleavage followed by the RT release. A relatively high RT activity associated with HML.2 GH purified on sucrose cushion might be predominantly attributed to RT in viral capsids captured by shedding MVs. This explains why the RT activity of HML.2 GH was reduced about 25 times, when dissolved pellet from gradient fraction was tested, while HML.2 Tera-1 RT activity was reduced only 2.5 times.

Afterwards, glycosylation of HML.2 GH and Tera-1 Env precursors and structural glycoproteins were examined. By Western blot analyses, it was shown that total lysate of GH cells contained significantly more cleaved precursors, compared to Tera-1. As shown above, this was a result of a high expression of several HML.2 loci in GH cells. Deglycosylation of viruses was performed after purification on sucrose cushion, since, after Dgs, the amount of SU was hardly detectable. Molecular mass of SU was estimated as 61 kDa. After PNGase F treatment, the molecular mass of SU was decreased to 42 kDa. It appears that the glycosylation profile of a reconstructed HERV-K113 Env reported previously [35] was close to that of HML.2 from teratocarcinoma cell lines. After deglycosylation of the HML.2 GH and Tera-1, besides p26-TM, a truncated protein of 24 kDa was revealed. Since cloning and sequencing did not show expression of a shorter TM, it was presumed that truncation occurs at the C-terminus of TM protein at a post-translational level. Two candidate motives for the furin cleavage were found at the N-terminus of TM (Figure 2d). It cannot be excluded that TM cleavage occurs in endosomes during SU-TM complexes delivery or later on in virus particles by viral protease. The role of p24-TM needs to be investigated.

To compare the density of attached carbohydrates, we introduced “index of glycosylation” (IG). The density of glycosylation is achieved by dividing protein length (in amino acids) by the number of putative glycosylation sites:

IG = L/n,(1)

IG—index of glycosylation (frequency of glycosylation sites per number of amino acids); L—protein length in amino acids (a.a.); n—putative number of carbohydrate attachment sites.

Depending on HML-2 loci, the lengths of SU protein varied from 415 a.a. to 465 a.a. and, accordingly, the IG diverged from 51 a.a. to 58 a.a. Ectodomain of TM is 168 a.a. long. Thus, the IG of TM ectodomain is 42 a.a. (Table 1). The density of HML.2 TM ectodomain glycosylation is the same as that of gp41TM ectodomain of HIV-1. Thus, it is possible to conclude that HML.2 Env from examined viruses are more densely glycosylated than most of the exogenous betaretroviruses (Table 1). In fact, the density of the HML.2 ectodomain glycosylation was equal to that of HIV-1. Dense glycosylation plays the role of a shield for the neutralization-sensitive TM regions, like the membrane proximal external region (MPER) in gp41 HIV-1 [46,47]. In this regard, it cannot be excluded that such «sensitive site(s)” were present in TM of the HML.2 founder virus.

The following stage of our work was focused on the morphology of virus particles. Earlier HML.2 from stimulated teratocarcinoma cells were examined by TEM using the ultrathin section and negative staining of cells [12,14,29,31]. These investigations demonstrated the release of bald virions with immature “doughnut like” (toroidal) capsids.

The HML.2 particles from gradient fractions with a buoyant density 1.16 g/cm^3^ were analyzed by negative staining and ultrathin section TEM. Negative staining revealed vesicles of different size and free bald virus-like particles of ~100 nm in diameter with amorphous capsids (Figure 2(a1)).

There are three principal classes of extracellular vesicles: exosomes (ES, <100 nm), microvesicles (MVs, 100–1000 nm) and apoptotic bodies (AB, mostly >1 µm) [39]. We performed a brief analysis of vesicles that might co-precipitate with HML.2 GH after ultra-centrifugation. To disclose the repertoire and parameters of vesicles in cell supernatant, filtration through the 0.45 µm membrane, before gradient centrifugation, was not performed. Pallets from the gradient fraction of HML.2 GH were analyzed by negative staining TEM. Aggregates composed of small <100 nm (likely ES) and larger vesicles of 100–300 nm in diameter (likely MVs) were disclosed. Bald virus-like particles of ~100 nm with condensed and amorphous capsids where found attached to extracellular vesicles (Figure 3(a2)). It is worth mentioning that MVs exert their biological functions through interactions with recipient cells and after membrane fusion events deliver their molecular cargo to those cells [48].

Further on, the HML.2 particles were examined by ultrathin section TEM. The repertoire of discovered virus particles was diverse, but bald particles with amorphous capsids were predominant (Figure 3(b1)). Structural similarity of HML.2 GH and HML.2 Tera-1 capsids was disclosed (Figure 3b). Particles with condensed or partially condensed capsids of 50–70 nm and ~6–8 nm long SP represented a minority (Figure 3(b4,b5) left). Finally, several particles with presumed TM stumps of 2–4 nm long were identified (Figure 3(b5) right). Evidently, detection of such small structures is difficult to achieve. However, the amount of such particles might be significant, as HML.2 TM was regularly detected by Western blot analyses. The rarity of virions with SP shown by TEM explains why detection of SU in HML.2 by sensitive Western blot analyses was problematic.

The morphological analyses revealed aberrant HML.2 capsid assembly. Bald particles with amorphous capsids were predominant. There might be several causes for such capsid assembly. First, it seems likely that weak non-covalent interactions between SU and TM in HML.2 might be weaker than expected. Decrease of interaction force between SU and TM might be caused by a short deletion(s) in TM, as shown for gp22 of MPMV [49]—or else it might be associated with point mutations, as shown for gp41TM HIV-1. Such mutations in TM significantly increase detachment of SU and by that reduces viral infectivity by ~90% [50]. The appearance of mutation(s) “critical” for the TM-SU interactions cannot be excluded in the genome of the HML.2 progenitor. Such mutations might still be present. Second, predominance of amorphous capsids might be caused by proviruses that express truncated Gag. During assembly, truncated Gag can “compete” with complete Gag and by that perturb proper capsid assembly. Another possibility is a deficiency of a full size vRNA [51], or a large excess of cellular RNA, which can interfere with assembly. Impaired CA–CA interaction, as a result of “restrictions” possessed by teratocarcinoma cells, cannot be excluded as well. A model of HML.2 capsid construction was established using cryo-EM [52]. The model shows the ability to form capsid with the same architecture as exogenous viruses.

Besides free particles, we spotted HML.2 GH association with exosomes, presumably MVs. A significant number of observed vesicles contained virus-like cargo, as budding MVs can pinch off fragments of plasma membrane [39] at the location of possible HML.2 assembly. Viral capsids and even assembled viral particles might represent the cargo (Figure 3(c6–c8)). Interestingly, particles with condensed capsids were also noted inside the MVs. This argues in favor of possible maturation of HML.2 inside these vesicles. As a rule, MVs deliver relatively small cellular elements composed of active molecules including microRNA, mRNA and DNA, proteins and peptides. How efficient the delivery of large elements like HML.2 might be remains to be determined. MVs utilize the cell entry system combined tetraspanins and peripheral phosphatidylserine, which interact with densely glycosylated cell receptors Tim 1 or Tim 4 [53]. The cellular cargo of MVs is complex [54], and each element of deposited cargo can be implicated in variable cellular processes [55]. A massive and lasting release of MVs with HML.2 cargo might turn on innate and adaptive immune processes in target cells. Along these lines, it was shown that about 60% of male patients with germ cell tumors mount an antibody response against the HML.2 Gag precursor, which declined after tumor removal [56]. It was also demonstrated that HML.2 TM can trigger the IL-6 and IL-10 release [57], while HML.2 by itself can break tolerance and induce immune responses [58]. Thus, we assumed that the HML.2 cargo can induce deregulation and promote diverse immune reactions in recipient cells. It remains to be determined whether viral cargo can trigger a “cytokine storm”, similar to that provoked by respiratory [59,60] and herpes viruses [61]. It should be emphasized that incorporation and transport of HIV-1 regulatory proteins (but not assembled particles) by extracellular vesicles was described [62].

Observed diversity of shapes and morphology of HML.2 capsids suggested that viruses with different parameters might be present in the pool. However, analyses of distribution of viral proteins and vRNA profile did not show well-defined evidence of HML.2 GH diversification. Performed studies revealed CA, TM and SU proteins and vRNA in fractions of the gradients. While the highest amount was detected in fractions of buoyant density 1.16 g/cm^3^, it should be noted that detection of SU glycoprotein was challenging. To obtain a weak signal, very sensitive detection methods and extended exposure of the membrane to a film were required.

Next, we analyzed vRNA packaged in examined HML.2. Previously, the expression of HML.2 type 1 provirus in Tera-1 cell lines and in particles was examined by two groups [63,64]. Both found that >90% of the virus particles that contained vRNA sequences originates from the type 1 provirus HERV-K101 (position 22q11.21). This provirus encodes a full-length *gag* gene, but not *env*, and therefore can provide the Gag precursor for particle assembly. In Tera-1 cells, eight transcriptionally active HML.2 proviruses were detected, and the relative cloning frequency of HERV-K108 (7p22.1) cDNA *env* was as high as 20.9% [63]. The HERV-K108 (7p22.1) encodes a functional Env protein [38].

We focused on type 2 *env* genes as they are coding for functional Env and Rec proteins. The 460 bp *env* amplicons were cloned and sequenced. Analysis of 25 clones from HML.2 Tera-1 demonstrated their identity or close proximity to HERV-K108 *env*. The repertoire of transcripts packaged in HML.2 GH was more complex. Sequencing of 25 clones revealed four members of the HML.2 family: HERV-K109 (9 clones), HERV-K108 (6 clones), HERV-K115 (4 clones) and HERV-K41 (4 clones) and two clones with recombinant *env*. Thus, diverse vRNA in HML.2 from teratocarcinoma cell lines were present. Extrapolating these results to events in vivo indicates that the HML.2 expression in tumors of similar genesis might be radically different. Detection of HERV-K 115 *env* sequence in an HML.2 GH pool might be of special interest, as the virus has a complete genome. Whether viral particles with condensed capsid and SP (Figure 3(b4,b5)) were derivatives of HERV-K115 or they were assembled using elements from different proviruses remains to be determined. Lack of HERV-K 115 Env incorporation into SIV or MLV pseudotypes showed previously [6] does not necessarily mean that the virus cannot be fully assembled.

The last part of the study was devoted to valuation of the HML.2 release from cells. The estimation was based on quantification of CA molecules in cell supernatants. The goal was to approximate the number of viruses that might be secreted by teratocarcinoma cells in vivo and compare it with HIV-1 release from infected lymphocytes. If not counting protein decay, the amount of CA released from GH cells cannot exceed 56,700 CA molecules or 27 capsids/virions per day. However, a significant number of Gag complexes were trapped inside MVs, thus the real amount of free virions might be significantly lower. To better appreciate the level of the HML.2 production, we compared it with lymphoid cells infected with HIV-1. It was shown that one lymphocyte from chronically infected patient can produce about 1 × 10^4^ virus particles in 24 h [65]. That amount is 370 and 1670 times higher than estimated maximal production rate of GH and Tera-1 cells, respectively. It has to be mentioned that the majority of virions that according to estimation might be produced per day and per cell on average are largely devoid of SU, as shown by TEM and Western blot analyses. However, TM might be present as stumps, as illustrated by TEM and by Western blot analyses. We emphasize that CA/virion quantification presented here is an approximation and serves only as a reference point.

## 4. Materials and Methods

### 4.1. Cells and Cellular Lysates

Human teratocarcinoma cell lines Tera-1(ATCC^®^ HTB-105) and GH [13], and human embryonic kidney cell line HEK293T (ATCC CRL-3216), were maintained in DMEM supplemented with 10% heat-inactivated fetal bovine serum (FBS), 1% penicillin–streptomycin and L-glutamine. Cell lines were tested for mycoplasma contamination using a MycoSensor PCR assay kit (Agilent Technologies, Santa Clara, CA, USA) and were found to be mycoplasma free. For lysate preparation, cells were washed four times with phosphate-buffered saline (PBS) and were lysed in NP40 buffer (1% NP-40; 150 mM NaCl; 50 mM Tris-HCl pH 8.0) containing protease inhibitor cocktail Complete (Roche, Mannheim, Germany). After 10 min on ice, the lysates were centrifuged at 10,000× *g* for 10 min. Supernatants were kept frozen at −80 °C.

### 4.2. Antibodies

The following primary antibodies against HML.2 were used: rabbit anti-p27-CA produced in our laboratory, mouse monoclonal antibodies anti-HML.2 TM (HERM 1811-5, Austral Biologicals, San Ramon, CA, USA) and rabbit anti-HML.2 SU peptide GNQTLETRDRKPFYC (Gene Script, Piscataway, NJ, USA). The reactivity of antibodies was tested using recombinant HML.2 viruses. Secondary antibodies used in this study: anti-rabbit IgG-HRP conjugate, anti-mouse IgG-HRP conjugate and anti-goat IgG-HRP conjugate all were from Dako Laboratories (Glostrup, Denmark).

### 4.3. Virus Isolation, Sucrose Density Gradient Centrifugation (Dgc)

Cell supernatants (250–350 mL) were centrifuged at 1000× *g* and 3000× *g* for 12 min to eliminate cells and cellular debris, respectively. This was followed by filtration through a 0.45 µm membrane filter (Merck Millipore, Burlington, MA, USA). The supernatants were either directly applied on a 20% sucrose cushion on TN buffer (10 mM Tris-HCl, 150 mM NaCl, pH 7.6) or centrifuged at 120,000× *g* for 1 h 30 min at 4 °C and then applied a dissolved pellet on a 20% sucrose cushion. After centrifugation at 120,000× *g* for 1 h 45 min, obtained pellets were dissolved in TN buffer to achieve a final 2000 times concentrate. Sucrose density gradient (20–60%) centrifugation was performed in 13 × 51 mm or 14 × 89 mm centrifuge tubes (Beckman Coulter, Palo Alto, CA, USA) at 160,000× *g* for 5 h at 4 °C. Fractions of the gradient were diluted in TN buffer (1:5) and pelleted by centrifugation at 160,000× *g* for 1 h 30 min. Obtained pellets were diluted in TN buffer and were kept at −80 °C. Supernatant from HEK 293T cells was processed and concentrated as virus-containing supernatants, and it was used as a negative control.

### 4.4. Transfection of Cells and Purification of Recombinant HML.2 Particles

HEK 293T cells were transfected with a set of expression vectors pCRVI/Gag-PR-Pol [37] (a kind gift of Dr Paul Bieniasz), pcDNA3_oricoRec and pcDNA3_oricoEnvΔ659-699-V5 coding the HML.2 genes gag, pol, rec and envΔ, respectively. The V5 tag is 14 amino acids long. The design of vectors was reported previously [32,35]. The JSRV expression vector was kindly provided by James DeMartini (Fort Collins, CO, USA). Transfection was performed in 6-well plates, when cells were at 50% of confluence (5 × 10^5^ cells). Four µL of TransIT-293 transfection reagent (Mirus Bio LLC, Madison, WI, USA) per 1 µg of plasmid and 0.5–1 µg of each plasmid per well were applied. Medium was changed 6 h post transfection. Two days after transfection supernatant was harvested, centrifuged and filtered as described (Section 4.3). Particles from cell-free supernatant were concentrated by ultracentrifugation through a 20% sucrose cushion. Virus proteins were detected by direct gels staining with Roti-Blue quick (Carl Roth GmbH, Karlsruhe, Germany) and by Western blot analyses.

### 4.5. Western Blotting and Dot Blot

Protein electrophoresis was performed in precast Tris-Glycine 4–20% gradient gels (ServaGel TG PRIME, Heidelberg, Germany) using SDS Tris-Glycine sample buffer (Novex, Life technologies, Carlsbad, CA, USA). Gels were calibrated using PageRuler Plus pre-stained protein ladder (Thermo Scientific, Waltham, MA, USA) and VisiBlot Standard I (Serva, Heidelberg, Germany), indicated in the text as M1 and M2, respectively. Proteins were transferred onto 0.2 µm membrane (Protran BA83, Whatman GmbH, Dassel, Germany) at 45 V for 2 h. Membranes were blocked with 6% skimmed milk (Carl Roth, Karlsruhe, Germany) in PBS with 0.1% Tween 20 (blocking solution) for 2 h at room temperature or overnight at 4 °C. Membranes were incubated with antibodies for 2 h at room temperature and washed in PBS with 0.1% Tween 20 (washing buffer). Then, the membranes were incubated for 1 h 30 min with secondary antibodies diluted in blocking buffer 1:10 K, washed five times with washing buffer and immune complexes were visualized with Pierce ECL Plus Western blotting substrate (Pierce, Rockford, IL, USA), or SuperSignal WestPico Plus (Thermo Scientific, Waltham, MA, USA). Blots were exposed to Amersham Hyperfilm ECL (GE Healthcare Limited, Pollards Wood, UK) or CL-XPosure Film (Thermo Scientific, Mortsel, Belgium). Dot blot assay was performed on 0.2 µm Protran BA83 membrane. One µL of diluted pellet from each gradient fraction was loaded on the strips. The membrane was dried for 15 min at room temperature. Then, it was stained with Fast-blue (Carl Roth, Karlsruhe, Germany) for 10 min and washed for 30 min in distillate water.

### 4.6. PNGase F Treatment

Deglycosylation experiments were performed using cytoplasmic lysates of GH and Tera-1 cells, and virions purified by centrifugation through the 20% sucrose cushion. Examined samples (8–10 µL) were treated with PNGase F according to the protocol of the supplier (New England Biolabs, Ipswich, MA, USA). Treated proteins were analyzed by Western blotting.

### 4.7. RNA Extraction, RT-PCR and RT-qPCR

Viral RNA (vRNA) was extracted using RNeasy mini kit and RNase-free DNase set, as recommended by the supplier (Qiagen, GmbH, Hilden, Germany). Quantification of RNA was performed employing NanoDrop spectrometer ND-1000 (PEQLAB, VWR Darmstadt, Germany). Extracted specimens of vRNA were immediately used in reverse transcriptase (RT)-PCR. Primers for the PCR and qPCR were selected using the IDT package (https://eu.idtdna.com/pages (accessed on 11 November 2018)). The amplification was performed using SensiFast Probe No-ROX one-Step kit (Bioline GmbH, Luckenwalde, Germany). The following primers were used for amplification (GenBank JN675068 served as reference): Primers for HML.2 env amplification: Forward (6615–6634) 5′-GCT GAC GCA GTT AGC TAC AA; Reverse (7074–7054) 5′-ATA ATT TAC CCG TGG CCT GAG. Primers for 5′TM-LTR amplification: Forward (8160–8181) 5′- GAC GCC ATC TACAGG GAA GA; Reverse (8647–8628) 5′-CAT GTT TCA GAG AGC ACG GG. Primers for 3′SU-LTR amplification: Forward (7772–7797) 5′-GCC ATC CAT CCA TAT TTT GGC TGA AG; Reverse (8647–8628) 5′-CAT GTT TCA GAG AGC ACG GG. 

RT-qPCR analysis of the fractions was performed using Luna universal One-step RT-qPCR kit, according to the recommendations of the supplier (New England Biolabs, Inc., Ipswich, MA, USA). Primers and probe for RT-qPCR env amplification: Forward (6688–6710) 5′-CAA TGG TGG TAA GTC TCC CTA TG; Reverse (6788-6769) 5′-CCA TGT GAC TGC CCG AAT TA; Probe (6737–6760) 5′-FAM-TAC CAA CTG GGC CTA TGT GCC TTT-BHQ. Primers and probe for RT-qPCR gag amplification (GenBank AY0379281): Forward (1628–1649) 5′-CCA TCA GAG TCT AAA CCA CGA G; Reverse (1728–1711) 5′-GGC GGT TGG GTC TTA TTT TC; Probe (1669–1694) 5′-FAM-AGG TCA GGT GCC CGT AAC ATT ACA AC-BHQ. Serial tenfold dilutions of a plasmid containing cloned full size HERV-K113 (pBSK-oriHERV-K113) were used as a reference [66].

### 4.8. Reverse Transcriptase Assay

The product-enhanced reverse transcriptase (PERT) assay has been used to detect HML.2 associated reverse transcriptase (RT) activity. The reaction was performed in a 96-well plate. Five µL of the virus containing samples purified on 20% sucrose cushion and 5 µL of virus samples from fractions (buoyant density 1.16 g/cm^3^) of the gradient were tested. The RT reaction was carried out for 20 min at 42 °C, followed by 45 cycles of PCR amplification. Parameters of the reaction were adopted from Vermeire et al. [67].

### 4.9. Cloning and Sequence Analysis

PCR amplicons were cloned into the pCR2.1-TOPO vector according to the protocol of the supplier (Thermo Scientific, Waltham, MA, USA). JM109 cells (Promega, Corp., Madison, WI, USA) were transformed and plated on LB/ampicillin agar for 16 h at 37 °C. Selected colonies were grown in LB/amp overnight at 37 °C. The plasmids were isolated using the PureYield plasmid mini preps system (Promega), tested for the inserts by digestion with Eco RI and sequenced using BigDye terminator kit V.3.1. (Applied Biosystems, Foster City, CA, USA). Sequences were analyzed using LaserGene Version 10 (DNASTAR, Inc., Madison, WI, USA) and Geneious package software (Biomatters Ltd., Auckland, New Zealand).

### 4.10. Transmission Electron Microscopy (TEM)

Preparation of samples for negative staining TEM was performed as described [68]. Briefly, particle suspensions from the main sucrose gradient fraction obtained by ultracentrifugation were fixed with 2% paraformaldehyde in 0.05 M HEPES buffer, adsorbed on a sample support and contrasted with uranyl acetate. Samples were examined with a transmission electron microscope (JEM-2100, JEOL Corp. Tokyo, Japan) operated at 200 KV, and images were recorded with a CCD camera and 2048 × 2048 pixels.

For ultrathin sections, virus suspensions were fixed as mentioned above and further concentrated by ultracentrifugation using a Beckman Coulter airfuge (Beckman Coulter, Life Sciences, Indianapolis, IN, USA). Therefore, a small volume (10 µL) of the virus suspensions was mixed with PBS (80 µL), a gold colloid suspension (15 nm cationic gold; 20 µL), glutaraldehyde (2.5%; 10 µL) and filled into polyallomer tubes (240 µL volume; Beckman Coulter, Life Sciences, Indianapolis, IN, USA) pre-filled with a low-melting point agarose gel (0.5%, 30 µL). After ultracentrifugation at 119,000× *g* for 10 min, the supernatant was discarded and 100 µL of liquid 3% low-melting point agarose was added on top of the virus pellet. Subsequently, the agarose was solidified on ice which fixed the pellet to the agarose gel. The pellet was localized at the surface of the gel bloc by the red coloration from the concentrated gold particles, and the gel region was extracted with a scalpel. To prevent the pellet from dispersal, the surface of the extracted gel piece was surrounded by a thin layer of 3% low-melting point agarose. Afterwards, gel pieces with the pellet were post-fixed with 1% osmium tetroxide, 0.1% tannic acid and 2% uranyl acetate, before they were dehydrated in ethanol and embedded in epon resin as described previously [68]. Thin sections (60–70 nm) were stained with 2% uranyl acetate and 0.1% lead citrate and inspected with a transmission electron microscope (Tecnai Spirit, FEI Company, Hillsboro, OR, USA) operated at 120 kV. Images were recorded with a CCD camera at 1360 × 1024 pixels.

### 4.11. Software

The NCBI Blast program was used for the database search. Parameters of PCR primers were selected and analyzed using the OligoAnalyzer 3.1 (Integrated DNA technologies, Coralville, IA, USA). Sequences were aligned using Lasergene Version 10 (DNASTAR, Inc., Madison, WI, USA) and Geneious package (Biomatters Ltd., Auckland, New Zealand) software. The image processing program (ImageJ, US National Institutes of Health, Bethesda, MD, USA) was used to compare protein bands intensity and ExPasy NetNGlyc 1.0 package was used to predict N-glycosylation sites. The ProP 1.0 Server (https://services.healthtech.dtu.dk/ (accessed on 15 February 2020)) was used to predict furin cleavage sites.

### 4.12. Sequences Deposited to the GenBank

HML.2 Tera-1 cloned sequences. Twelve clones were identical with HML.2 (K108) (GenBank acc. #AF164614.1). Two identical clones: #8 and #25 (M2230783); two identical clones #12 and #21 (M2230782). Three identical clones #33, #14, #22 (MZ330696); three identical clones #30, #19, #24 (MZ330697). A single clone: #1 (MW890762).

HML.2 GH cloned sequences. Clone #32 is identical with HML.2 (K108) (GenBank acc. #AF164614.1). Three identical clones #4, #34, #42 (M2230780) and two identical clones #33, #41 are close to HML.2 (K108). Four clones #7, #31, #38 and #40 are identical with HML.2 (K109) (GenBank acc. #AF164615.1); two identical clones #25 and #26 (MZ292718); two identical clones #18 and #29 (MZ292719). Single clones: #3 (MZ330695), #5 (M2230781), #12 (MZ292716), #20 (MZ2300778), #21 (MZ2300778), #23 (MZ292717), #37 (MZ292720). Recombinant clones: #10 (MW811393) and #14 (MW811394).

## 5. Summary and Conclusions

The most important results are summarized below:

- As a consequence of active expression of several proviruses, GH cells produced 4–5 times more viruses, compared to Tera-1 cells. It is likely that SU and TM of HML.2 are completely glycosylated. Truncated TM variant (p24-TM), a low SU content and reduced RT activity were disclosed in examined HML.2.
- Bald virions with amorphous capsids were the most common morphological variants in HML.2 pools. Virus particles with SP and condensed (collapsed) capsids were rarely detected.
- GH cells actively release MVs. During budding, MVs can capture fragments of plasma membrane with HML.2 elements at different stages of assembly. Judging from the TEM images, maturation of virus particles inside MVs may take place. The release of MVs from Tera-1 cells was insignificant.
- We assume that MVs with HML.2 elements upon delivery to target cells can trigger cell signaling systems, promote inflammation and autoimmune diseases.
- Six different HML.2 transcripts including two recombinant variants were packaged into virus particles from GH cells. However, HML.2 Tera-1 contained only HERV-K108 *env* transcripts and similar sequences.
- It was assessed that, in 24 h, one GH cell can produce ~27 virions, while Tera-1 cell ~6 virions, whereas human lymphocytes chronically infected with HIV-1 release about 1 × 10^4^ virions.

To conclude, a comprehensive analysis of human endogenous retroviruses HML.2 GH and Tera-1 was performed. Concentration of the cell supernatant by 2000 times and subsequent virus purification made it possible to detect viruses and advance research on HML.2. In gradient fractions with a buoyant density 1.16 g/cm^3^, CA, TM and a low amount of SU were detected. Both HML.2 demonstrated a low RT activity and insignificant vRNA content. Clonal analyses of HML.2 Tera-1 revealed exclusively HERV-K108 type 2 *env* genes, while HML.2 GH contained *env* genes from four proviral loci and also two recombinant env sequences. Besides free viral particles, extracellular vesicles (presumably MVs) that contained HML.2 viral structures at various stages of assembly were disclosed.

HML.2 viruses from teratocarcinoma cell lines, because of low expression level, aberrant morphology of capsids, a minimal amount of SU and a low RT activity, are unlikely to infect cells. Indicated shortcomings explain why previous attempts to infect human and animal indicator cells failed [14]. However, the possibility of recombination with exogenous viruses can lead to the formation of replication competent forms [2,36]. Here, we proposed another mechanism of HML.2 implication in cell pathogenesis. As it is shown, entire HML.2 particles or its fragments might be captured during shedding of MVs. This “forced cooperation” with MVs permit to deliver viral cargo to recipient cells. So, MVs to some extend can play a role of “translocation chaperon” for HML.2. We think that such delivery might trigger cell signaling systems, promote inflammation, autoimmune diseases and other pathologies. The effect on cells might be proportional to the frequency of deliveries. There are several reports concerning transportation of viral elements by MVs (for a review, see [69]), but, to our knowledge, this is the first demonstration of MVs as a possible delivery vehicle for HML.2.

## Figures and Tables

**Table 1:** Predicted glycosylation sites (gls) in HML.2, exogenous beta-retroviruses and HIV-1.

Viruses	Total gls	SU gls	TM gls	TM Ectodomain (a.a./gls)	TM Ectodomain IG (a.a./gls)
HERV-K108 (HML.2)	11	7 *	4	168/4	42
HERV-K109 (HML.2)	11	7 *	4	168/4	42
HERV-K113 (HML.2)	11	7 *	4	170/4	~42.5
HERV-K115 (HML.2)	11	7	4	170/4	~42.5
HERV-K108 (HML.2)	11	7	4	170/4	~42.5
JSRV	9	6	3	171/3	57
ENTV	8	5	3	170/3	~56.6
MMTV	5	3	2	168/2	84
MPMV	11	10	1	167/1	167
HIV-1	31	24	7 (-3) **	170/4	~42.5

* Excluding glycosylation site in single peptide and/or “NPT” motives in SU. ** Excluding three gls in endodomain.

## Data Availability

Data are available upon reasonable request.
